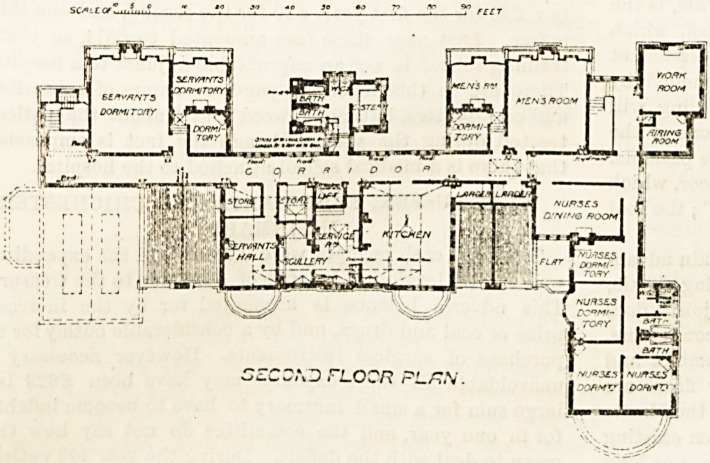# Jewish Home for Incurables, Tottenham

**Published:** 1901-11-23

**Authors:** 


### The Institutional Workshop.

### JEWISH HOME FOR INCURABLES, TOTTENHAM.

The architects state that the primary object was to obtain an institution which should be strictly domestic in character and feeling, and which should not impress the occupants with an idea either that they were living in a hospital or of being subjected to any strict discipline. In criticising the plans it is necessary to bear this in mind, as it probably formed part of the instructions given to the architects; but we doubt whether the mere form of a building can have much effect in obtaining the results aimed at in the minds of the patients. At all events it should not be allowed to interfere with recognised canons of hospital construction when these are essential to the well-being of the patients themselves.

The building is divided by a corridor which runs almost north and south. At the north end of this corridor is the hall, with an entrance for the staff and for tradesmen. On the west are the matron's room, a six-bedded dormitory, a one-bedded room with bath-room attached, a sanitary block correctly cut off from the main by efficiently cross-ventilated passages, another one-bedded room and another six-bedded dormitory. At the south end of the corridor there is a slope permitting Bath chairs to be easily taken out of or into the hospital. On the east side of the corridor are two six-bedded rooms and two living rooms, the latter being divided from each other by a servery, a store, and a lift. The living rooms have fine circular bays. At the north-east angle is a large room used as a committee room and an entertainment room. It has a separate entrance and a corridor leading to the hall, and a secretary's office adjoins it. The first floor is almost the same as the ground floor, the only difference being that two single-bedded rooms are placed over the secretary's office and the end of the committee room corridor. The second floor is given up to the kitchen and its offices, and to accommodation for the nursing and domestic staff. The six-bedded dormitories facing east are not carried up to the second floor.

If we except the six-bedded dormitories on both sides of the corridors, and on both floors, it may be said that the general arrangements are good, are easily workable, and seem to have been carefully thought out. These dormitories are, however, of faulty design. Two on each floor have windows only in one end, and the others have, in addition to these, only one window in one side. There can, therefore, be no efficient cross-ventilation by means of the windows, and this is one of the most important points of hospital construction at the present day. Indeed, the larger dormitories of any institution for the sick should be first planned, and the other parts should be subservient to these dormitories, and should interfere as little as possible with the conditions named. Here the opposite principle seems to have been followed, and the dormitories are wedged between other rooms instead of having three of their sides facing the open air. We do not overlook the fact that there are ventilators and extraction flues; but we do not think these can atone for the drawbacks we have mentioned.

The building is heated by open fire-places on the Teale principle, and there are also hot-water radiators worked from central boilers. This combination of open fires and hot-water radiators is beyond doubt the correct system for an institution of this nature.

Arrangements for extinguishing fire and for the escape of patients in the event of fire have been carried out, and the building is fitted up with electric bells, speaking tubes, and telephones.

The grounds are extensive, and have been laid out as gardens for the use of the patients. The architects are Messrs. H. H. and M. E. Collins. The cost of the building is not stated.

## Figures and Tables

**Figure f1:**
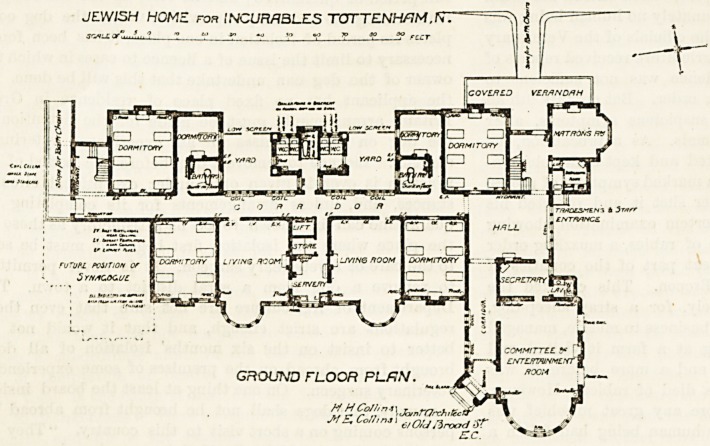


**Figure f2:**
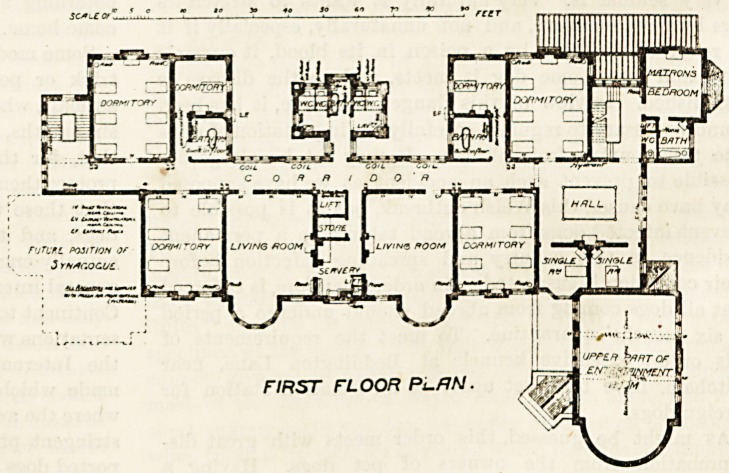


**Figure f3:**